# Reliability and statistical power: Conceptual background and practical implications

**DOI:** 10.3758/s13428-026-03068-z

**Published:** 2026-07-13

**Authors:** Attila Krajcsi

**Affiliations:** Cognitive Psychology Department, Institute of Psychology, ELTE Eötvös Loránd University, Izabella Utca 46, 1064 Budapest, Hungary

**Keywords:** Statistical power, Reliability, Reliability paradox

## Abstract

In the behavioral sciences, robust phenomena having large effect sizes and related statistical power may be unreliable. This relation is in contrast to the view that both statistical power and reliability depend similarly on measurement error. In this view, robust but unreliable phenomena can be considered as a reliability paradox. The mathematical relation and possible independence of statistical power and reliability have been discussed in the statistical literature for decades. A common misconception related to the relationship between statistical power and reliability is that absolute reliability (measurement error) is often confounded with relative reliability (ratio of the true score variance to the total score variance). The present work reviews and discusses how measurement error and individual differences influence the relative reliability and robustness of a phenomenon. It discusses why relative reliability and robustness are partly related and why they are generally independent. Following these considerations, it summarizes how measurement error and group heterogeneity can be potentially manipulated to optimize the reliability and robustness of a measured phenomenon.

## The reliability paradox

In the behavioral sciences, many robust phenomena or effects (i.e., effects that are easy to demonstrate or replicate) are not reliable when measured with indices such as test–retest correlation and split-half correlation. Robustness with low reliability can be seen as a paradox (Hedge et al., 2018; Kucina et al., 2023; Overall & Woodward, 1975, 1976).

For example, in the Stroop task, participants have to name the color of the ink of a word, where the word is the name of a color (Stroop, 1935). In the task, while the word is not relevant, it influences the responses; for instance, blue ink is harder to name when the stimulus is the word *red* than when it is the word *blue*. This effect is robust in the sense that it is easy to demonstrate that the interference index differs significantly from zero, even in relatively small samples (Ebersole et al., 2016). In statistical terms, the standardized effect size is relatively large, so that even with a relatively small sample size, the effect can be demonstrated; in other words, the appropriate hypothesis test has large power even with a relatively small sample. However, this robust effect may be unreliable. For example, when the effect is measured twice, the test–retest reliability is often low (Hedge et al., 2018). Many other well-known cognitive effects can be easily observed as statistically significant, while their reliability is relatively low (Hedge et al., 2018; Kucina et al., 2023; Rouder et al., 2023; Schuch et al., 2022).

It may be surprising for many researchers when robust cognitive phenomena that can be routinely demonstrated show low reliability. It is often assumed that robustness depends on the noise in the measurement, and reliability measures this noise; therefore, it seems paradoxical that robust phenomena are not reliable. This seeming contradiction is also known as the reliability paradox (Hedge et al., 2018).

The seeming contradiction behind the paradox is not only an empirical observation. Mathematically, for example, it has been demonstrated that unreliable gain (difference) scores may have appropriate power when an experimental manipulation-induced change should be detected (Overall & Woodward, 1975, 1976). From a statistical viewpoint, this paradox is equivalent to the problem of how the statistical power of hypothesis tests comparing central tendencies (e.g., a *t*-test) is related to the reliability of the dependent variables (Fleiss, 1976; Hopkins & Hopkins, 1979; Nicewander & Price, 1978, 1983; Overall & Woodward, 1975, 1976; Sutcliffe, 1980; Williams et al., 1995; Williams & Zimmerman, 1980, 1989, 1989; Zimmerman & Zumbo, 2015). According to the widespread and seemingly reasonable assumption of many researchers, if reliability reflects the precision of the measurement or the amount of noise in the data, then lower noise (higher reliability) should lead to a smaller variance in the data, which in turn leads to better power in hypothesis tests investigating the expected value. Therefore, high reliability means low noise, which should mean higher power for hypothesis tests. With these assumptions, one may ask how it is possible to observe the high power of the hypothesis test and the low reliability of the variables for a phenomenon at the same time.

The aim of the present paper is to explain and resolve this seeming contradiction in a concise and accessible way. This paradox, or several key aspects of it, has been discussed and partly explained and resolved both from the viewpoint of practical research (Byers-Heinlein et al., 2022; Dang et al., 2020; De Schryver et al., 2016; Hedge et al., 2018) and from the statistical viewpoint (Fleiss, 1976; Hopkins & Hopkins, 1979; Nicewander & Price, 1978, 1983; Overall & Woodward, 1975, 1976; Parsons, 2018; Sutcliffe, 1980; Williams & Zimmerman, 1980, 1989; Williams et al., 1995; Zimmerman & Zumbo, 2015). The present description summarizes the key points of these explanations while extending and refining some of these statements to provide a more comprehensive and—hopefully—intuitive account of the key concepts. The present work also connects practical research and mathematical viewpoints. Using a more appropriate view of the relations between reliability and power in correlational and central tendency comparison analyses, one may plan and evaluate studies more precisely and efficiently.

In the present review, first, we clarify the often-intermingled distinctive meanings of reliability (Section "Two different meanings of reliability"). Second, we summarize some key components of the central tendency comparison analyses (Section "Robustness of empirical phenomena"). Third, we investigate the role of the measurement noise and the individual differences in reliability and central tendency tests (Sections "Opposing roles of individual differences in reliability and robustness" and "Mathematical independence of reliability and robustness"). Then, we discuss whether robustness and reliability are really related in research practice (Section "Are robust phenomena unreliable?"). Finally, practical considerations are summarized that help researchers plan their empirical studies more efficiently (Sections "The role of reliability and robustness in correlational and central tendency comparison studies" and "Practical implications").

## Two different meanings of reliability

The paradox stems partly from ambiguity related to the concept of reliability. Reliability can be either absolute or relative, and arguments are not always explicit about which reliability concept is considered. Here, we summarize the main features of absolute and relative reliability that are essential in understanding the conceptual relations behind the reliability paradox.

Conceptually, in its general sense, reliability is the precision of the measurement or, equivalently, the lack of noise in the measurement. For this reason, reliability may be expressed as the amount of noise in the measurements (in statistical terms, the measurement error) (Lindsay & Mather, 2022; Luck, 2019). For example, in a reaction time measurement, one may determine the extra variance of the noise that is added to the actual processing time and its variance. In this example, reliability is considered in an absolute way.

**Fig. 1:**
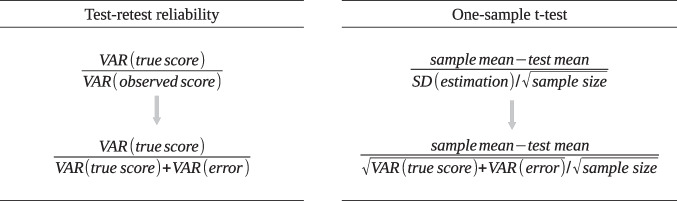
Key components of the test–retest reliability and one-sample *t*-test. The upper row displays the original formulas, while the lower row displays the formulas, highlighting the role of the measurement noise (i.e., measurement error) and the individual differences (i.e., variance of the true score)

However, when reliability is measured in research practice, most often some correlation-based or functionally equivalent indices are used, such as the test–retest correlation, the split-half reliability (where a test is split into two halves, for example, taking even and odd items or trials, and the correlation of the two halves is calculated), Cronbach’s alpha, which in some circumstances can be considered as the mean split-half correlations of all possible splits of a set of items or trials (Cronbach, 1951), or some of the intraclass correlation (ICC) indices (McGraw & Wong, 1996). This kind of reliability index is theoretically motivated by classical test theory. In classical test theory, it is assumed, and therefore defined, that a measured score comprises the true score and the measurement error. Given this assumption, reliability is defined as the variance of the true score relative to the total score variance, where the total score variance is the sum of the variance of the true score and the variance of the measurement error. In practical research, the mathematical term of true score variance refers to the individual differences in the participants. Statistically, this can be described as $reliability=\frac{true\;score\;variance}{total\;score\;variance}=\frac{true\;score\;variance}{true\;score\;variance\;+\;error\;variance}$ (see also the left side of Fig. 1). Note that this definition of reliability is equivalent to the correlation of the indices measured twice (Revelle, 2011).

Regarding the ratio of the true score to total score variances, one may think about it as a standardized index of the measurement noise, where the noise is standardized by the size of the individual differences.^1^ Conceptually, this is similar to other well-known standardized effect sizes. This standardization is reasonable when one is interested in the seriousness or relevance of the noise in the given measurement and not in the noise in an absolute sense. For example, 30 ms noise variance is large if individual differences show 5 ms variance, but it is small if individual differences show 200 ms variance. Thus, this reliability reflects the relative size of the noise. This reliability index has the advantage that, because the amount of noise is expressed in the size of individual differences, the reliabilities of various variables and studies can be compared, and the importance or seriousness of the noise is expressed in a more standardized way. This standardized reliability index is a relative index as opposed to absolute reliability (i.e., absolute measurements of the measurement noise).

When calculating reliability, relative reliability indices are usually used. However, it is critical to highlight that this is only one way to measure or express reliability: As mentioned above, reliability can be expressed not only in this relative or standardized form but also in an absolute way (Byers-Heinlein et al., 2022; Lindsay & Mather, 2022; Luck, 2019; Luck et al., 2021).

It is essential to see that while, conceptually, reliability is often considered as the noise in the measurement (absolute reliability), in practical calculations, the relative reliability indices are applied. This difference is often ignored. As explained below, from the viewpoint of the reliability paradox, it is fundamental to differentiate between absolute reliability (measurement noise or measurement error) and relative reliability (standardized indices that are used most often in practical calculations) (Nicewander & Price, 1983).

Note that the present work refers to these two kinds of reliabilities as absolute and relative reliabilities (Lindsay & Mather, 2022); however, other terms are also used, such as measurement error and reliability (Byers-Heinlein et al., 2022; De Schryver et al., 2016), precision and reliability (Luck, 2019; Luck et al., 2021), or reliability and reliability coefficient (Nicewander & Price, 1983; Sutcliffe, 1980). The terms absolute and relative reliabilities may be more transparent: there are no ambiguities about which term someone refers to, and they reflect key differences between the two kinds of reliabilities.

Importantly, relative reliability has another critical property that is fundamental to understanding the reliability paradox. As discussed above, this reliability is a standardized index where the measurement error (absolute reliability) is contrasted with the variability of the true score (i.e., by the size of the individual differences). This means that relative reliability indices depend on both the measurement error and the variability of the true score. In other words, relative reliability depends not only on absolute reliability but also on individual differences. On the one hand, increasing or decreasing the noise leads to lower or higher relative reliability, respectively—this is in line with the conceptual assumption that reliability is the precision (or noisiness) of the measurement. On the other hand—and this is one of the keys to resolving a common misconception—increasing the true score variance leads to higher relative reliability, and decreasing this variability leads to lower relative reliability (reliability row in 1). True score variance is the individual differences: high variance means a heterogeneous group, while low variance means a homogeneous group. Consequently, a more heterogeneous group leads to higher reliability, and a more homogeneous group means lower reliability. For example, the reliability of an index can be lower in a university student population than in a same-age population with any educational level because university students are more homogeneous than the more general group. This also means that, given the same amount (or distribution) of noise, changing the population changes the reliability index. This role of the true score variance is inconsistent with the intuitive concept of absolute reliability: Relative reliability depends not only on the absolute reliability (measurement noise) but on individual differences. The role of the size of the individual differences is unintuitive because individual differences have nothing to do with reliability in a more general sense, that is, with absolute reliability.

**Table 1:** Influencing factors for relative reliability and power of a hypothesis test (here, one-sample *t*-test)

Measured indices	Influencing factors	Change in the index after increasing the factor
Relative reliability	Measurement noise (absolute reliability)	↓ Larger noise decreases relative reliability
	True score variance	↑ Larger true score variance increases relative reliability
Power of a one-sample *t*-test	Measurement noise (absolute reliability)	↓ Larger noise decreases the power
	True score variance	↓ Larger true score variance decreases the power

## Robustness of empirical phenomena

While estimating robustness, empirical phenomena are often detected with hypothesis tests that investigate mean differences. Here, the simple example of the one-sample *t*-test is taken, but the present reasoning can be extended to similar central tendency comparison hypothesis tests, such as other *t*-tests, analyses of variance (ANOVAs), or nonparametric tests (Nicewander & Price, 1978; Williams et al., 1995).

In a one-sample *t*-test, the question is whether the measured sample may come from a hypothetical population that has a mean with a specific value. For example, in the case of the Stroop effect, it is tested whether the measured interference index in the sample could come from a population where the index is 0 (i.e., the null hypothesis assumes the lack of the effect). Obviously, because of random sampling, practically, the mean of a sample is never the same as the mean of the population the sample comes from. Therefore, the more technical question behind the one-sample *t*-test is how probable it is that the sample with the measured mean (or a more extreme value) can come from a hypothetical population. This answer depends on three numerical properties. The test statistic of the one-sample *t*-test (the *t*-value) is calculated as $\frac{sample\;mean\;-\;hypothetical\;population\;mean}{SDestimation/\sqrt{\text{sample size}}}$ (see also the top right part in Fig. 1). A more extreme *t*-value means that it is less likely that the sample came from the hypothesized population. In this test, according to the formula, there are three factors that may demonstrate a deviation from the hypothetical population. A larger difference between the sample mean and the hypothetical population mean (i.e., larger raw effect size), smaller standard deviation, and larger sample size make it more unlikely that the sample comes from the hypothetical population.

The robustness of a phenomenon, when detected with a hypothesis test investigating means, is the power of the hypothesis test: when the effect exists, and we want to demonstrate it with a hypothesis test, robustness is the chance of having a significant result. The power of a hypothesis test, in general, depends on the alpha, the standardized effect size, and the sample size. In a one-sample *t*-test, the standardized effect size is the ratio of the raw effect size (the difference between the sample mean and the hypothesized population mean) to the standard deviation of the sample. The role of these components is also visible in the formula of the *t*-value above.

From the viewpoint of the reliability paradox, the role of the standard deviation should be understood. The standard deviation of the sample influences the power of the test. Considering the standard deviation in terms of classical test theory, the standard deviation of the sample (or equivalently, the sample variance) has two sources: the true score variance (i.e., individual differences) and the measurement noise (i.e., the absolute reliability) (bottom right part in Fig. 1). Considering these components’ effect on the power (or the *t*-value), decreasing any source of these variances increases the chance of having a significant result. In other words, decreasing the measurement error variance (absolute reliability) or decreasing true score variance (individual differences or heterogeneity) increases power (power row of Table 1).

## Opposing roles of individual differences in reliability and robustness

**Fig. 2:**
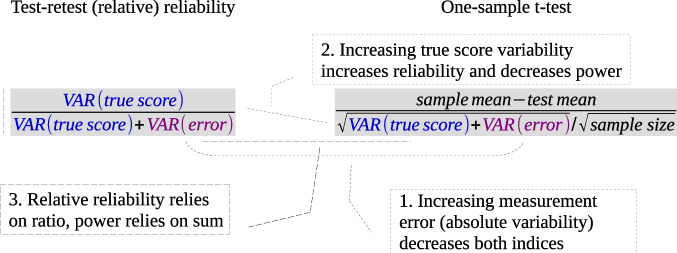
Factors influencing reliability and statistical power

At this point, one critical reason why robust phenomena may have low reliability in some cases may become apparent. On the one hand, larger absolute reliability (measurement noise) decreases both the correlation-based relative reliability and the power of the *t*-test (Table 1 and point 1 in Fig. 2). On the other hand, the true score variance of the sample (individual differences) has opposing effects on the two indices. Larger true score variance (individual differences) makes the measurement noise look relatively small (i.e., the relative size of the noise is smaller when the true score variance is larger; this is because the reliability index is standardized where the noise is standardized with individual differences); therefore, larger true score variance increases the relative reliability index. However, in the one-sample *t*-test, a larger true score variance increases the overall variance (or, equivalently, the standard deviation), which decreases the power (Table 1 and point 2 in Fig. 2). In other words, while large individual differences are good for the relative reliability index, they are bad for the power of the one-sample *t*-test (Byers-Heinlein et al., 2022; May & Hittner, 2003; Williams et al., 1995).^2^

It is clear that measurement noise (absolute reliability) in itself is not sufficient to specify the relative reliability or the power of the phenomenon-detecting hypothesis test, even if larger measurement noise (smaller absolute reliability) in itself leads to lower relative reliability and lower power. It is also clear why reasoning about the relation of relative reliability and power based only on measurement noise (absolute reliability) is misleading. These conceptual roles also partly clarify the misconception about the relation between relative reliability and the power of a hypothesis test. While researchers appropriately conceptualize the role of the measurement noise (absolute reliability) in those indices, they may ignore the fact that true score variance (individual differences) also plays a role in those indices, and the direction of this variance has opposing effects on the two indices (Table 1 and point 2 in Fig. 2).

This opposing role of individual differences in reliability and robustness also aligns with the reliability paradox. When experimental tasks and related phenomena are selected to be robust, phenomena with relatively small individual differences are preferred so that the power of the central tendency testing analyses is relatively high, while the small individual differences lead to relatively small relative reliability (Hedge et al., 2018).

## Mathematical independence of reliability and robustness

As discussed above, large individual differences are good for reliability, but they are bad for the robustness of the empirical phenomena (point 2 in Fig. 2; Hedge et al., 2018). However, this is not the complete picture of the role of individual differences in relative reliability, and the power of the hypothesis test and an additional detail should be considered. As mentioned above, while noise has a similar effect on both relative reliability and robustness, individual differences have opposing effects (Table 1; points 1 and 2 in Fig. 2). However, the effects of noise and individual differences are combined differently in relative reliability and robustness. As explained above, in the relative reliability index, the reliability depends on the measurement noise standardized with the individual differences: The ratio of the true score variance to the measurement noise variance is considered. However, in the one-sample *t*-test, the true score variance and the measurement error variance are summed up, and this sum is used as the variance of the sample. Therefore, while in the relative reliability, the ratio of the measurement noise and individual differences is used, in the one-sample *t*-test, their sum is applied (point 3 in Fig. 2; Zimmerman & Zumbo, 2015). This is another fundamental reason why the test–retest reliability index and the power of the hypothesis tests have a complex and hard-to-understand relation.

**Fig. 3:**
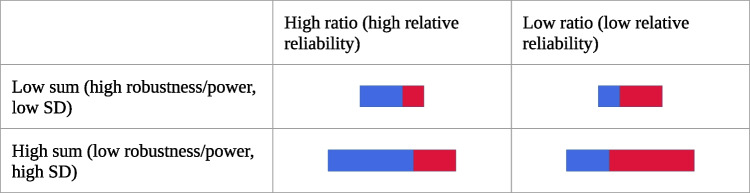
Illustrating the independence of robustness (rows) and relative reliability (columns). In the color bars, blue represents true score variance (i.e., individual differences), and red represents measurement noise

This difference has another fundamental consequence: the ratio of the measurement noise and individual differences is mathematically entirely independent of the sum of the measurement noise and individual differences. Mathematically, the ratio in itself is not informative about the sum, and vice versa. For example, if one knows that the ratio of two numbers is 2, the sum of the numbers can be anything, such as 3 (as with 2 + 1), 6 (4 + 2), or 4.5 (3 + 1.5). Relatedly, it is possible that the sum of the measurement noise and individual differences is small (the robustness is large), and the ratio may show either the relative majority of true score variance (high relative reliability) or the relative majority of the measurement noise (low relative reliability) (see the low sum row in Fig. 3). Similarly, even if the sum is large (low robustness), either the true score variance or the measurement noise can be the relative majority of the whole variance (see the high sum row in Fig. 3). In other words, knowing only the robustness or power, one cannot determine the relative reliability, or knowing only the relative reliability, one cannot determine the robustness or power. (See related visualizations in Fig. 1 in Byers-Heinlein et al., 2022, and Fig. 1 in Parsons, 2018.) This also means that while in relative reliability the key question is what the ratio of the true score variance to the measurement noise is, the sum is irrelevant. In contrast, in the central tendency hypothesis tests, the ratio is irrelevant but the sum should be considered.

In line with this argument, it was already argued in the 1970 s that low-reliability gain (difference) scores can be robust (Overall & Woodward, 1975, 1976), or conversely, reliability and robustness may correspond in other circumstances (Fleiss, 1976). It was clarified in the same decade that robustness and reliability are independent mathematically, and the previous seemingly contradictory results had depended on specific additional assumptions about fixed parameters; for example, it was falsely assumed that individual differences could not be changed (Nicewander & Price, 1978, 1983; Williams & Zimmerman, 1989; Williams et al., 1995).

This independence of robustness and relative reliability also shed light on why the reliability paradox is not a paradox in a strict mathematical sense. The paradox assumes that reliability is the noisiness of the measurement. It also assumes that noisy measurement contributes to low power in hypothesis tests, and if a phenomenon is robust (i.e., the power of the relevant hypothesis test is large), then the reliability must also be high. According to this reasoning, it is surprising to see robust but unreliable phenomena. However, this argument does not differentiate between absolute and relative reliabilities. According to a more appropriate reasoning, the two kinds of reliabilities must be differentiated. The noisiness of the measurement is the absolute reliability. Power depends on the sum of absolute reliability and individual differences, while relative reliability depends on the ratio of absolute reliability and individual differences; consequently, power and relative reliability are independent unless other common components are known. Therefore, it is not surprising to observe robust but unreliable phenomena, and the seeming reliability paradox is not a paradox at all.

## Are robust phenomena unreliable?

Above, it was first noted that the opposing role of individual differences in power and relative reliability may explain why robust phenomena may be unreliable (Hedge et al., 2018). Later, it was also described that power and relative reliability are mathematically independent, and the reliability paradox is illusory. So, is there a reliability paradox or not?

In fact, there are two independent questions here. The first question is whether there can be robust but unreliable phenomena. The answer is clearly supportive. Mathematically, robustness and relative reliability are independent in the sense that knowing only one of them, one cannot specify the other index (Nicewander & Price, 1978; Williams & Zimmerman, 1989; Zimmerman & Zumbo, 2015). The seeming paradox only emerges if absolute and relative reliabilities are confounded. Also, empirical works have reported many robust but unreliable phenomena (Enkavi et al., 2019; Hedge et al., 2018; Rouder et al., 2023; Schuch et al., 2022). Therefore, robust but unreliable phenomena are entirely in line with the notions of statistical power and absolute and relative reliabilities.

A second question is whether robust phenomena are usually unreliable, that is, whether robust phenomena are not only possible, but they appear frequently (Hedge et al., 2018). Indeed, many robust phenomena are unreliable (Enkavi et al., 2019; Hedge et al., 2018; Krajcsi & Szűcs, 2022; Rouder et al., 2023; Schuch et al., 2022). It is also reasonable to argue that many experimental tasks are selected for robustness (while investigating only the group means); therefore, paradigms showing small individual differences are preferred; consequently, the tasks selected for robustness are usually unreliable (Hedge et al., 2018). On the other hand, there are robust phenomena that are also reliable (Enkavi et al., 2019; Krajcsi & Szűcs, 2022).

Reformulating this second question, one may ask whether there is a correlation between robustness and relative reliability when investigating phenomena that are frequently described in research. Currently, there are no available results to answer this question. A correlation between robustness and relative reliability in popular paradigms can be investigated only if the set of tasks is comprehensive or representative. However, studies demonstrating robust and unreliable tasks often use a potentially biased subsample of psychological phenomena: these studies often choose tasks for which gain (i.e., difference) scores should be calculated (Enkavi et al., 2019; Hedge et al., 2018; Krajcsi & Szűcs, 2022; Rouder et al., 2023; Schuch et al., 2022), where gain scores are known to have lower reliability than their component scores (Overall & Woodward, 1975; Williams & Zimmerman, 1996). A more representative selection of phenomena may show a smaller or no relationship between robustness and reliability. In other words, robust tasks may or may not be unreliable, but the studies that demonstrate unreliable tasks utilize robust tasks that use gain score indices with typically low reliability, whose task selection may be biased. Future research may reveal whether such a correlation is observable.

## The role of reliability and robustness in correlational and central tendency comparison studies

As described so far, robustness and relative reliability are, strictly speaking, or mathematically, unrelated. Importantly, they have different roles in correlational and mean comparison analyses. The present section summarizes how reliability and robustness influence correlational and central tendency comparison analyses.

Regarding the correlational (and, more generally, regression) analyses, it has long been known in the methodological literature that non-perfect reliability attenuates (decreases) the observed correlation (Spearman, 1904, 1910; find simple demonstrations of the attenuation in Fisher, 2014; Osborne, 2019). It may be easy to understand that any measurement noise makes it difficult to observe the true relation between variables. Overall, whenever the relative reliability is lower than 1 (practically, this is always the case), the observed correlation is lower than the true correlation, and the lower the relative reliability, the larger the attenuation of the observed correlation. Mathematically, $observed\;{correlation}_{xy}=true\;{correlation}_{xy}\sqrt{{reliability}_{x}\cdot {reliability}_{y}}$. Therefore, the relative reliabilities of the variables in a correlation analysis directly influence the observed correlation.

In terms of the power of related hypothesis tests about the correlational/regression analyses (note that this is the power of the correlational/regression analysis, not the power of the central tendencies test discussed so far above), because the observed correlation is lower than the true correlation, a larger sample is needed for a given power compared to a fictional situation in which the true correlation could be measured (e.g., see Table 5 in Hedge et al., 2018). Importantly, the lower the reliabilities of the variables are, the lower the observed correlation can be, and the lower the power is for a given sample size. In summary, the reliability of the relevant variables influences the observed effect size of the correlation or regression, and for this reason, it influences the power of the related correlational hypothesis test as well (Table 2).

While the relative reliability influences the observed correlation and the related power (i.e., the power of a hypothesis test that checks the correlation), it cannot directly influence the central tendency comparison analysis and its power (note again that this is the power of the central tendency hypothesis test, not the power of the correlation test discussed in the previous paragraph.), as discussed above (Table 2).

Regarding the central tendency comparison studies, statistical power is conceptually the robustness of the finding. Larger power can be reached with a smaller sample variance. From the viewpoint of the present discussion, sample variance is the sum of the true score variance and the measurement error variance. However, as discussed above, relative reliability relies on the ratio of these two components; therefore, relative reliability in itself does not directly influence the robustness of the central tendency comparison (Table 2).

Overall, relative reliability is relevant for correlational analyses but not central tendency comparison analyses. Inversely, the power of the comparison hypothesis test is relevant for central tendency comparison analyses but not in correlational analyses (Table 2) (Byers-Heinlein et al., 2022).

**Table 2:** The effects of the reliability and power of the comparison test on correlational and central tendency comparison analyses

	Analysis type	
Relevant properties	Correlational analyses	Central tendency comparison analyses
Relative reliability	Has effect	No effect
Power of the comparison hypothesis test	No effect	Has effect

## Practical implications

On the basis of the above considerations, the practical implications are relatively straightforward. Critically, the recommended practices also depend on the analyses a researcher wants to apply. Note that these recommendations mainly discuss the role of the factors the present work considers: the role of the measurement noise and sample variance (i.e., individual differences).

1. If the task is already reliable (measured with relative reliability) and/or robust (i.e., has the appropriate power for a comparison test), then it fulfills its goal and provides an appropriate base for the analyses; therefore, no modifications are needed.
2. When the task is not reliable/robust, try to improve the reliability/robustness.
  - If the task is not reliable and/or robust, then independently of the analysis type, the noise can be decreased, e.g., by increasing the number of trials/items of the task (Table 3, first row; Nicewander & Price, 1978). Theoretically, the noise can be decreased by controlling for the environmental factors, such as avoiding disturbance during the measurement or decreasing the measurement error of the devices; however, some results suggest that these factors may have a limited effect compared to the effect of the number of trials (Lindskog et al., 2013).
  - If the task is not reliable and/or robust, depending on the type of analyses, one may aim to change the size of the individual differences. For correlational studies, the variance should be increased (e.g., by choosing a more heterogeneous group; Table 3, second row), which will increase the relative reliability and decrease the attenuation, although this will decrease the power for central tendency comparison tests. For central tendency comparison, the variance should be decreased (e.g., by choosing a more homogeneous group), which will increase the power of the comparison, although this will decrease the relative reliability (Byers-Heinlein et al., 2022; Hedge et al., 2018; Nicewander & Price, 1978). In general, the variance of the group (i.e., homogeneity) can be changed by changing the target population. For example, university students are more homogeneous than the same age group with any educational background; children of a narrower age range are more homogeneous than those of a wider age range; including older participants or participants with developmental issues may increase the heterogeneity.
3. If the task is not reliable and/or robust and cannot be reasonably improved by changing the noise and/or the sample variance, then, depending on the circumstances, different approaches may be used. For example, increasing the sample size increases the power of a hypothesis test. Alternatively, one may consider changing the task or paradigm.
  - Finally, if nothing else works, then sometimes it must be acknowledged that the given research question cannot be investigated in the way it was initially considered.

**Table 3:** Recommended modifications when the task robustness/reliability is not sufficient

	Analysis type	
Factor of action	For correlational analyses	For central tendency comparison analyses
Noise	Decrease	Decrease
Individual differences	Increase	Decrease

## Conclusions

Reliability and statistical power are two key components of statistical analyses and, more generally, in planning a study and interpreting the results. One common misconception is conflating measurement error (absolute reliability) and commonly used standardized reliability indices (relative reliability). Another key point to understanding the relationship between reliability and statistical power is understanding how measurement error and individual differences influence reliability and power. Finally, it is also essential to see that correlational and central tendency comparison analyses rely on distinct features of the data. Understanding these key points makes planning and interpreting studies more straightforward, and researchers can modify the measurement noise and homogeneity of the sample more efficiently.

## Data Availability

Not applicable.

## Footnotes

1 Technically, a standardized noise would be the ratio of the noise variance to the true score variance. However, the reliability correlation is the ratio of the true score variance to the observed score variance. Still, it is easy to find the relation between the correlation and the standardized noise. The reciprocal of the reliability correlation (i.e., 1/reliability) is $\frac{true\;score\;variance+error\;variance}{true\;score\;variance}\;=\;1\;+\;\frac{error\;variance}{true\;score\;variance}=standardized\;noise\;+\;1.\;Therefore, standardized\;noise=\frac{1}{reliability}\;-\;1$.

2 Here, only a simple hypothesis test is considered; however, see additional details for other tests (such as other *t*-tests, ANOVAs, or nonparametric tests) in the statistical literature (e.g., May & Hittner, 2003; Nicewander & Price, 1978, 1983; Williams & Zimmerman, 1989).
